# The Role of Diet in Modulating Inflammation and Oxidative Stress in Rheumatoid Arthritis, Ankylosing Spondylitis, and Psoriatic Arthritis

**DOI:** 10.3390/nu17091603

**Published:** 2025-05-07

**Authors:** Daria Kupczyk, Rafał Bilski, Łukasz Szeleszczuk, Katarzyna Mądra-Gackowska, Renata Studzińska

**Affiliations:** 1 Department of Medical Biology and Biochemistry, Faculty of Medicine, Ludwik Rydygier Collegium Medicum in Bydgoszcz, Nicolaus Copernicus University in Toruń, 24 Karłowicza St., 85-092 Bydgoszcz, Poland; 2 Department of Organic and Physical Chemistry, Faculty of Pharmacy, Medical University of Warsaw, 1 Banacha Str., 02-093 Warsaw, Poland; lukasz.szeleszczuk@wum.edu.pl; 3 Department of Geriatrics, Faculty of Health Sciences, Collegium Medicum in Bydgoszcz, Nicolaus Copernicus University in Toruń, 9 Skłodowskiej Curie Str., 85-094 Bydgoszcz, Poland; katarzyna.madra@cm.umk.pl; 4 Department of Organic Chemistry, Faculty of Pharmacy, Collegium Medicum in Bydgoszcz, Nicolaus Copernicus University in Toruń, 2 Jurasza Str., 85-089 Bydgoszcz, Poland; rstud@cm.umk.pl

**Keywords:** rheumatoid arthritis, ankylosing spondylitis, psoriatic arthritis, diet, gut microbiota, oxidative stress, inflammation, Mediterranean diet, omega-3 fatty acids, polyphenols

## Abstract

Rheumatic diseases such as rheumatoid arthritis (RA), ankylosing spondylitis (AS), and psoriatic arthritis (PsA) are chronic autoimmune disorders characterized by persistent inflammation and oxidative stress, leading to joint damage and reduced quality of life. In recent years, increasing attention has been given to diet as a modifiable environmental factor that can complement pharmacological therapy. This review summarizes current evidence on how key dietary components—such as omega-3 fatty acids, fiber, polyphenols, and antioxidant vitamins—affect inflammatory pathways and oxidative balance. Special emphasis is placed on the Mediterranean diet, low-starch diets, and hypocaloric regimens, which have shown potential in improving disease activity. The gut microbiota emerges as a critical mediator between diet and immune function, with dietary interventions capable of restoring eubiosis and strengthening the intestinal barrier. Additionally, this paper discusses challenges in the clinical implementation of diet therapy, the need for personalized nutritional strategies, and the importance of integrating diet into holistic patient care. Collectively, findings suggest that dietary interventions may reduce disease activity, mitigate systemic inflammation, and enhance patients’ overall well-being.

## 1. Introduction

Rheumatic diseases, including rheumatoid arthritis (RA), ankylosing spondylitis (AS), and psoriatic arthritis (PsA), pose a significant medical and social challenge [1]. These chronic autoimmune disorders are characterized by persistent inflammation and considerable consequences for patients’ quality of life [2]. The primary mechanism underlying these diseases involves dysregulation of the immune response, leading to the release of proinflammatory cytokines such as tumor necrosis factor alpha (TNF-α), interleukin-6 (IL-6), and interleukin-1 beta (IL-1β). These cytokines exacerbate the inflammatory process and contribute to tissue damage, particularly in articular cartilage and bone [3].

Oxidative stress, defined as an imbalance between the production of reactive oxygen species (ROS) and the body’s antioxidative capacity, also plays a crucial role in the pathogenesis of these disorders. ROS can activate inflammatory pathways, such as NF-κB, and induce damage to lipids, proteins, and DNA, thereby amplifying the inflammatory state. This oxidative imbalance is closely associated with the progression of rheumatic diseases and their systemic complications, including atherosclerosis and metabolic syndrome [4,5].

In rheumatic diseases, inflammation is a key element of pathogenesis. One of the main pathways is the IL-23/IL-17 axis, where IL-23 promotes the differentiation of Th17 lymphocytes, and IL-17A stimulates the production of proinflammatory cytokines, chemokines, and adhesion molecules. At the same time, the activation of the NF-κB pathway by pathogen-associated molecular patterns (PAMPs) leads to the production of TNF-α and subsequent inflammatory mediators. An important role is also played by NLR receptors (including NLRP3), which activate cytokines such as IL-1β and IL-18, intensifying the inflammatory response. In addition, angiogenesis, mainly regulated by VEGF, supports the development of inflammatory and bone changes in tissues. Excessive oxidative stress enhances these processes by activating inflammatory pathways and inducing cell apoptosis. The interaction of these mechanisms leads to chronic inflammation, tissue damage, and pathological remodeling of joint structures [4,5].

Current treatment strategies include pharmacotherapy based on disease-modifying antirheumatic drugs (DMARDs), which encompass both conventional and biological agents (e.g., TNF-α inhibitors), as well as nonsteroidal anti-inflammatory drugs (NSAIDs). However, their efficacy is often limited by adverse effects and variable patient responses. Consequently, growing attention is being directed toward nonpharmacological interventions—most notably dietary modifications—that can influence inflammatory and oxidative processes [6,7,8,9,10,11,12].

Diet is a key environmental factor in the pathogenesis of rheumatic diseases. Evidence suggests that appropriate dietary habits may exert protective effects by reducing inflammation, limiting oxidative stress, and improving gut microbiota function. Particular interest has focused on dietary patterns such as the Mediterranean diet, low-starch diets, and hypocaloric regimens, which provide anti-inflammatory and antioxidant components, including omega-3 fatty acids, fiber, polyphenols, and antioxidant vitamins [13,14,15,16,17].

The gut microbiota, which plays a pivotal role in immune system modulation, is also closely linked to diet. Gut dysbiosis—characterized by reduced levels of probiotic bacteria and an overrepresentation of proinflammatory species—is frequently observed in patients with RA, AS, and PsA. Dietary interventions can alter the composition of the gut microbiota, promoting the production of short-chain fatty acids (SCFAs) that exhibit anti-inflammatory properties and support the integrity of the intestinal barrier [18,19].

The aim of this review is to provide a comprehensive overview of the role of diet in modulating inflammatory and oxidative processes in RA, AS, and PsA. Specifically, this article examines the mechanisms of action of key dietary components, their effects on the gut microbiota, and the findings of clinical studies assessing the effectiveness of dietary interventions. Additionally, we propose how dietary strategies may complement pharmacological therapy and highlight their potential benefits for patients.

## 2. Inflammation and Oxidative Processes in RA, AS, and PsA

Rheumatic diseases are characterized by a complex pathogenesis in which inflammatory processes and oxidative stress play a central role [4,20]. Elucidating these mechanisms is essential for identifying effective therapeutic strategies, including dietary interventions.

Inflammatory processes in rheumatic diseases are driven by immune system activation, encompassing both innate and adaptive responses. A key component is the overproduction of proinflammatory cytokines, such as TNF-α, IL-6, and IL-1β, which play a central role in initiating and amplifying inflammation. In RA, these cytokines stimulate synovial fibroblasts, macrophages, and T lymphocytes, leading to chronic synovitis, cartilage degradation, and bone destruction [21,22,23].

In AS, inflammatory processes are associated with activation of the IL-23/IL-17 pathway. IL-23, secreted by macrophages and dendritic antigen-presenting cells, promotes the differentiation of Th17 cells, which produce IL-17—a cytokine that exacerbates inflammation at enthesis sites and leads to pathologic new bone formation [24,25,26]. A hallmark of AS is the presence of the HLA-B27 haplotype, which exhibits unique properties, such as a tendency to misfold and form homodimers. This can intensify inflammation by activating killer-cell immunoglobulin-like receptors (KIRs) on NK cells and T lymphocytes [27,28,29].

Oxidative stress is a key pathogenetic factor in psoriasis and PsA. ROS generated by neutrophils and macrophages intensify inflammation, damage cellular structures, and activate pathways such as NF-κB [30,31]. In cutaneous psoriasis, ROS promote keratinocyte proliferation and induce vascular endothelial growth factor (VEGF) expression, contributing to angiogenesis within psoriatic lesions. In PsA, similar mechanisms can amplify inflammation in the synovium and periarticular tissues, leading to joint and bone damage [5,32,33,34].

As in RA and AS, gut dysbiosis is crucial in PsA. Patients with PsA show reduced gut microbiota diversity and an overabundance of proinflammatory bacteria, such as *Escherichia coli* [35]. Dysbiosis increases intestinal permeability, facilitating lipopolysaccharide (LPS) translocation into the bloodstream and triggering an inflammatory response. Clinical studies in patients with psoriasis and PsA have revealed correlations between the degree of dysbiosis and circulating levels of proinflammatory cytokines, including IL-17 and TNF-α [36,37].

Oxidative stress, arising from excessive production of reactive oxygen species, is instrumental in amplifying inflammatory processes. ROS induce damage to lipids, proteins, and DNA, thereby activating inflammatory pathways such as NF-κB and enhancing the secretion of proinflammatory cytokines. While high levels of ROS promote tissue damage and amplify inflammation, physiological ROS levels can exert regulatory effects, including the neutralization of reactive nitrogen species (RNS), thereby controlling the inflammatory response. Thus, antioxidants may exert their beneficial effects partly through the modulation of ROS and RNS levels [30,31]. Elevated levels of oxidative stress markers (e.g., malondialdehyde [MDA]) and reduced activity of antioxidant enzymes (e.g., superoxide dismutase [SOD] and glutathione peroxidase [GPx]) have been documented in RA, AS, and PsA [4,5]. Studies in animal models confirm that modulating the oxidative balance can reduce the severity of inflammatory responses [38].

## 3. Role of Histocompatibility Antigen in Ankylosing Spondylitis

HLA-B27, strongly associated with AS, contributes to its pathogenesis via several mechanisms, including the “arthritogenic peptide” hypothesis, endoplasmic reticulum misfolding, and homodimer formation. According to the arthritogenic peptide hypothesis, HLA-B27 presents bacterial or autoantigenic peptides that can activate autoreactive T cells [39,40]. Furthermore, HLA-B27 tends to accumulate in the endoplasmic reticulum, inducing ER stress and the activation of the unfolded protein response (UPR), which leads to the production of cytokines such as IL-23 and IL-17 [39,41]. In parallel, HLA-B27 homodimers on cell surfaces can stimulate proinflammatory pathways by interacting with KIR and LILR receptors [42,43].

## 4. Intersection of Psoriasis and Joint Inflammation

Psoriatic arthritis is an inflammatory autoimmune condition that combines psoriatic skin lesions with joint involvement. Its pathogenesis is tightly connected to the mechanisms underlying psoriasis, particularly the overactivity of the IL-23/IL-17 axis and oxidative stress, which drive chronic inflammation in both the skin and joints [44,45].

Central to the pathogenesis of both psoriasis and PsA is the hyperactivity of the IL-23/IL-17 axis. Dendritic cells in the skin and synovium secrete IL-23 in response to environmental and endogenous stimuli, such as tissue damage or alterations in the gut microbiota. IL-23 promotes the differentiation of Th17 lymphocytes, which produce IL-17—a cytokine that induces the expression of inflammatory mediators such as IL-6, TNF-α, and matrix metalloproteinases (MMPs). These mediators contribute to cartilage degradation, abnormal keratinocyte proliferation, and inflammatory changes in the skin. IL-17 is particularly important in PsA pathogenesis because it enhances neutrophil responses and promotes angiogenesis, thereby exacerbating both cutaneous lesions and joint inflammation. In addition, it activates dendritic cells in the synovium, creating an inflammatory loop that underlies persistent joint inflammation. In addition to Th17 lymphocytes, γδ T cells represent an important source of IL-17 in rheumatic diseases such as PsA and psoriasis. IL-23 not only promotes the differentiation of Th17 cells but also supports the expansion and activation of γδ T cells, which contribute to the inflammatory milieu [44,45,46].

In PsA, IL-17 also interacts synergistically with TNF-α to potentiate the inflammatory response, which is the basis for the efficacy of biological therapies targeting these cytokines [47,48].

## 5. Impact of Metabolic Factors and Diet in PsA

PsA, like psoriasis, is strongly associated with obesity and metabolic syndrome. Visceral adipose tissue acts as a reservoir for proinflammatory cytokines, such as leptin, TNF-α, and IL-6, which reinforce systemic inflammation. Adipocytes also produce ROS, exacerbating oxidative stress. Weight reduction through dietary interventions, such as hypocaloric diets, has been shown to significantly reduce disease activity in PsA patients, as demonstrated in clinical trials such as the DIETA trial [49,50,51].

A diet rich in omega-3 fatty acids, fiber, and antioxidants can modulate inflammatory and oxidative processes in PsA. Omega-3 fatty acids inhibit the IL-23/IL-17 pathway, while antioxidants (e.g., vitamin C and polyphenols) neutralize ROS, thereby reducing oxidative stress. Hypocaloric diets not only lower body weight but also reduce levels of proinflammatory adipokines and cytokines, such as IL-6, ultimately contributing to decreased disease activity [50,52].

## 6. The Role of Gut Microbiota in Inflammatory and Oxidative Processes

A unique, host-specific ecosystem known as the gut microbiota is located within the human gastrointestinal tract. This microbial community comprises bacteria, fungi, archaea, viruses, and other eukaryotes. The number and type of microorganisms vary along different segments of the gastrointestinal tract, influenced by factors such as pH, oxygen availability, intestinal transit time, and the secretory activities of various organs and tissues. The gut microbiota performs metabolic, trophic, and immunological functions, with the products of one biochemical pathway often serving as substrates for subsequent reactions. The metabolic activity of the microbiota includes the breakdown of undigested dietary residues and the maintenance of trophic functions. Moreover, gut microbes play an important role in modulating bone density and in synthesizing vitamins (notably B vitamins and vitamin K). Proper qualitative and quantitative proportions of gut microbiota enhance mineral absorption from food, and the microbiota also contributes to the production of free amino acids [53,54].

The gut microbiota plays a pivotal role in regulating the immune system [55]. Dysbiosis, defined by an increased abundance of proinflammatory bacteria (e.g., *Prevotella copri*) and a decreased diversity of probiotic species, is frequently observed in rheumatic diseases. Such dysbiosis can enhance intestinal permeability and promote the translocation of endotoxins, triggering an inflammatory response. Notably, a link between dysbiosis and the IL-23/IL-17 pathway activity has been reported in AS [56,57].

Recent studies have highlighted the crucial involvement of gut microbiota in the pathogenesis of numerous diseases. A balanced microbial composition, referred to as “eubiosis”, maintains homeostasis. In contrast, alterations in the microbiota induced by various environmental or host-specific factors can trigger a state of “dysbiosis”, often accompanied by disrupted gut barrier function. Dysbiosis has been associated with the pathogenesis of chronic inflammatory diseases [53,54]. Internal factors such as autoimmune disorders may compromise this barrier; hence, maintaining a healthy gut microbiota is critically important. Diet emerges as a significant modifiable factor in this context: the composition of the gut microbiota can change within as little as two days in response to dietary modifications. By shaping the gut microbial ecosystem through dietary measures, both local and systemic microbiota-driven functions can be modulated.

From a nutritional standpoint, optimal microbial balance in the gut enhances the efficient absorption of food-derived nutrients. A Mediterranean-style diet has been shown to induce favorable shifts in gut microbiota composition, largely due to an increased intake of vegetables, fruits, legumes—rich in vitamins, minerals, and dietary fiber—and fermented dairy products. Fiber and polyphenols stimulate the growth of commensal microbes, while limiting meat consumption further supports a healthier microbiome. Non-digestible polysaccharides, which are fermented in the large intestine, are among the main drivers of beneficial microbial proliferation.

Although the pathogenesis of rheumatic diseases has not yet been fully elucidated, studies indicate that the gut microbiome, elevated intestinal permeability, and aberrant immune responses may promote the development and progression of autoimmune conditions, including RA. In AS, gut barrier dysfunction can precede disease onset, underscoring the importance of preserving gut barrier integrity and functionality in autoimmune disorders [58,59,60].

In rheumatic diseases such as RA, AS, and PsA, significant alterations in gut microbiota composition—collectively termed dysbiosis—have been documented. Dysbiosis can amplify inflammatory and oxidative processes by disrupting the interplay between the gut microbiota and the immune system while also increasing intestinal permeability [58].

Under normal physiological conditions, the gut microbiota contributes to homeostasis through the production of SCFAs, including butyrate, propionate, and acetate, which are metabolites of dietary fiber fermentation. SCFAs exert robust anti-inflammatory effects by inhibiting the NF-κB signaling pathway and reducing the production of proinflammatory cytokines such as TNF-α and IL-6. Additionally, SCFAs strengthen the gut barrier by lowering epithelial permeability and curbing the translocation of bacterial toxins (e.g., lipopolysaccharides) into the bloodstream. LPS, a component of Gram-negative bacterial cell walls, can activate Toll-like receptors (TLRs) on immune cells, triggering an inflammatory cascade that further exacerbates rheumatic conditions [58,59,60].

Patients with RA, AS, and PsA often exhibit reduced levels of probiotic bacteria such as *Faecalibacterium prausnitzii* and *Bifidobacterium*, both critical for maintaining immunological homeostasis. Simultaneously, proinflammatory species (e.g., *Prevotella copri*, *Escherichia coli*) become more prevalent. This dysbiosis fosters increased gut permeability, enabling LPS translocation into systemic circulation. Elevated LPS levels can prompt T lymphocytes to differentiate into Th17 cells, which secrete IL-17—a cytokine central to the pathogenesis of AS and PsA. Animal model studies have demonstrated that heightened intestinal permeability and circulating LPS levels directly correlate with aggravated inflammation and activation of the IL-23/IL-17 signaling pathway in affected joints [60,61,62,63,64].

In AS, particular attention has been directed toward the presence of *Klebsiella pneumoniae*, linked to Th17 cell activation and IL-23 production by dendritic cells. IL-23 promotes Th17 cell differentiation, driving the secretion of inflammatory cytokines such as IL-17 and IL-22. These cytokines intensify inflammation at enthesis sites, culminating in chronic inflammation and pathological bone formation. Both animal and clinical studies indicate that specific shifts in gut microbiota correlate with disease activity and therapeutic outcomes [65,66,67].

In PsA, gut dysbiosis exacerbates cutaneous and articular symptoms. Patients with PsA have shown diminished microbial diversity and reduced SCFA levels. Insufficient SCFA production compromises gut barrier integrity, facilitating the translocation of endotoxins and escalating systemic inflammation. Furthermore, the gut microbiota can influence local inflammatory processes in the skin, thereby increasing keratinocyte proliferation and angiogenesis, hallmark features of psoriatic lesions [37,68].

Dietary strategies are of considerable importance in regulating microbiota composition and function. High-fiber diets support the proliferation of SCFA-producing probiotic species, bolstering the intestinal barrier and mitigating inflammation. Polyphenol-rich foods—such as fruits, vegetables, and green tea—promote microbial diversity while reducing the abundance of proinflammatory bacteria. Restricting saturated fats and simple sugars further diminishes TLR activation and bacterial endotoxin translocation, attenuating the inflammatory response. Clinical findings show that in AS and PsA patients, dietary regimens rich in fiber and prebiotics are associated with lower CRP and IL-6 levels, improved disease activity indices (including the Bath Ankylosing Spondylitis Disease Activity Index [BASDAI] and Disease Activity Score [DAS28]), and a reduction in pain [69,70,71,72].

These insights have sparked interest in exploring therapeutic probiotics. The commensal bacterium *Akkermansia muciniphila* is a promising candidate due to its capacity to strengthen the gut barrier, regulate host immunity, mitigate inflammation, and modify metabolic processes, effectively supplementing the daily diet with beneficial microbial components [73]. Given that RA can be precipitated by dysbiosis, it appears logical to complement dietary therapy with appropriately selected probiotics.

A summary of key information on gut microbiota in inflammatory conditions is provided in Table 1.

Prebiotic-containing foods have shown promising effects in modulating gut microbiota composition and, consequently, reducing inflammation in rheumatic diseases. Dietary prebiotics can promote the growth of beneficial bacteria such as *Faecalibacterium* and *Prevotella*, which are associated with anti-inflammatory effects through the production of short-chain fatty acids like butyrate. Studies have demonstrated that in conditions like rheumatoid arthritis and psoriatic arthritis, gut dysbiosis often coincides with decreased microbial diversity and an imbalance between proinflammatory and anti-inflammatory bacterial populations. By enriching the gut with prebiotics, it may be possible to restore microbial balance, enhance intestinal barrier integrity, and reduce systemic inflammation, offering a supportive strategy alongside conventional therapies [36,53].

## 7. The Importance of the Mediterranean Diet in Modulating Inflammation and Oxidative Stress

The Mediterranean diet (MD) is widely recognized as one of the most effective nutritional patterns for reducing inflammatory processes and oxidative stress. Its beneficial properties stem from a wealth of components with proven anti-inflammatory and antioxidant activities, including unsaturated fats, dietary fiber, polyphenols, and vitamins C and E [79,80]. Studies suggest that adherence to MD not only lowers inflammatory markers (e.g., CRP) but also improves the quality of life in patients with rheumatic diseases [81].

Olive oil, a fundamental component of MD, is rich in hydroxytyrosol and oleocanthal—compounds with potent anti-inflammatory effects. Oleocanthal acts as a natural inhibitor of cyclooxygenases (COX-1 and COX-2), thereby reducing the production of inflammatory mediators such as prostaglandins and leukotrienes. Hydroxytyrosol, in turn, neutralizes reactive oxygen species and protects cells from oxidative damage. Regular consumption of olive oil has been shown to decrease TNF-α and IL-6 levels in patients with RA [82,83,84,85].

Fatty fish such as salmon and mackerel, which are rich in omega-3 fatty acids, represent another crucial component of MD [86]. Omega-3 fatty acids reduce the production of proinflammatory eicosanoids, including prostaglandin PGE2 and leukotriene LTB4, while simultaneously increasing levels of anti-inflammatory eicosanoids [87]. These mechanisms lead to reduced Th17 lymphocyte activity and inhibition of osteoclast differentiation—effects particularly beneficial in RA and AS [88,89]. Polyphenols found in fruits and vegetables, such as resveratrol and catechins, inhibit the activation of inflammatory pathways like NF-κB [90].

Moreover, MD fosters the growth of beneficial gut bacteria, including *Lactobacillus* and *Bifidobacterium*, and stimulates the production of SCFAs. This supports the integrity of the intestinal barrier and limits endotoxin translocation [91]. Clinical studies have demonstrated that following MD for 12 weeks in patients with RA results in reduced CRP levels, improved DAS28 scores, and a decrease in the number of tender joints [92]. In AS and psoriatic arthritis, this dietary pattern lessens disease activity and slows joint damage progression, underscoring its potential role as an adjunct to pharmacotherapy [93,94,95].

## 8. The Significance of a Low-Starch Diet in Ankylosing Spondylitis

AS is a chronic inflammatory disease primarily affecting axial joints, leading to their ankylosis and restricted mobility. A low-starch diet has garnered particular interest regarding AS, given that its potential benefits go beyond mere caloric restriction. The hypothesis positing advantages of reduced starch intake is based on mechanisms involving the gut microbiota, the modulation of inflammatory pathways, and the reduced activity of bacteria such as *Klebsiella pneumoniae*, which plays a central role in AS pathogenesis [16,96,97].

The gut microbiota is a key component of the “gut–joint axis”, whose dysfunction may contribute to the development of AS. *Klebsiella pneumoniae* is of particular relevance, as increased abundance of this species has been observed in AS patients, correlating with heightened inflammatory activity and more severe clinical symptoms. This bacterium can provoke cross-reactive immune responses between its antigens and human antigens, potentially leading to autoimmunity. Starch—commonly found in many food products—serves as a primary energy substrate for *Klebsiella pneumoniae* [65,66,67,97]. Limiting starch intake reduces the availability of this substrate, thereby decreasing the bacterial population in the gut and diminishing its production of proinflammatory metabolites.

A low-starch diet also supports improved intestinal barrier function. In AS patients, gut dysbiosis is common and frequently associated with increased intestinal permeability, which in turn enables the translocation of lipopolysaccharides into the bloodstream. LPS stimulates TLR4 receptors on macrophages and dendritic cells, leading to the release of proinflammatory cytokines. By reducing starch consumption, the population of LPS-producing bacteria is lowered, thereby alleviating immune system activation and dampening inflammation [97,98].

In summary, a low-starch diet appears to be a promising strategy for complementing existing treatments for AS. Its potential arises from the ability to modulate the gut microbiota, enhance gut barrier function, and diminish the activity of key inflammatory pathways. Nonetheless, additional research is required to elucidate its long-term benefits and determine the optimal way to implement this dietary approach.

## 9. The Role of a Hypocaloric Diet in PsA

Psoriatic arthritis is an autoimmune disease in which joint inflammation is combined with skin symptoms characteristic of psoriasis. PsA is often associated with obesity and metabolic syndrome, which not only increase the risk of developing the disease but also worsen its course [5]. Excess body weight promotes chronic inflammation through the secretion of proinflammatory cytokines from adipose tissue, such as leptin, resistin, and TNF-α [99]. In this context, a hypocaloric diet that reduces body weight may play a key role in reducing disease activity.

Visceral adipose tissue acts as an active endocrine organ, producing adipokines that enhance inflammation. Leptin, the level of which increases with the amount of adipose tissue, stimulates the activation of Th17 lymphocytes and the secretion of IL-17, a key cytokine in the pathogenesis of PsA. Resistin, another adipokine, increases the expression of TNF-α and IL-6, which increase inflammation in the joints and skin [99,100,101]. Obesity also promotes oxidative stress, increasing the production of reactive oxygen species in adipocytes and immune cells. ROS exacerbate tissue damage and activate inflammatory pathways such as NF-κB, which further exacerbate symptoms [102,103,104].

A hypocaloric diet, by reducing body weight, reduces levels of proinflammatory adipokines and reduces systemic inflammation. In the DIETA trial, patients with PsA who followed a hypocaloric diet for 12 weeks had significant improvements in DAS28-CRP and reduced their number of swollen joints. These effects were seen regardless of the degree of weight loss, suggesting that changes in diet quality may have an independent effect on disease activity [50].

A hypocaloric diet also affects lipid and carbohydrate metabolism, which may limit the development of metabolic syndrome in patients with PsA. Improving insulin sensitivity and lowering triglyceride levels reduce the activation of inflammatory pathways in adipocytes and endothelial cells [105,106,107]. Moreover, reducing oxidative stress by limiting calorie intake may improve immune cell function and reduce ROS-induced damage [108,109,110]. A hypocaloric diet not only reduces inflammation and oxidative stress, but also improves the quality of life of patients with PsA. Clinical studies confirm that patients who follow this diet experience reduced fatigue and improved physical fitness. However, it is worth noting that the effectiveness of this diet depends on its quality, not only on a reduction in calorie intake. The inclusion of anti-inflammatory ingredients, such as omega-3 acids and polyphenols, may additionally increase its effectiveness in the treatment of PsA.

## 10. The Importance of Specific Dietary Components in Regulating Inflammation and Oxidative Stress

Diet therapy in rheumatoid diseases such as RA, AS, and PsA is based on the use of nutrients with proven anti-inflammatory and antioxidant properties. Omega-3 fatty acids, fiber, polyphenols, and antioxidant vitamins play a key role in reducing inflammation and neutralizing reactive oxygen species, which are significant factors exacerbating pathological processes in these diseases [72,111,112].

Omega-3 fatty acids, including eicosapentaenoic acid (EPA) and docosahexaenoic acid (DHA), have strong anti-inflammatory effects [113,114]. EPA and DHA are mainly present in oily fish such as salmon, mackerel, and sardines, as well as in chia seeds and linseed. Their mechanism of action is based on the regulation of eicosanoid production, which are key mediators of inflammation. EPA and DHA compete with arachidonic acid for the enzymes cyclooxygenase (COX) and lipoxygenase (LOX), reducing the production of proinflammatory prostaglandins series 2 (PGE2) and leukotrienes series 4 (LTB4). Instead, omega-3s promote the synthesis of anti-inflammatory eicosanoids series 3 (PGE3) and leukotrienes series 5 (LTB5) [115,116,117]. Omega-3s also modulate T cell function by reducing the activation of the IL-23/IL-17 pathway, which is key in AS and PsA. Additionally, EPA and DHA reduce the expression of inflammatory genes activated by NF-κB, which limits the production of TNF-α and IL-6 [118]. In clinical studies, omega-3 supplementation at doses of ≥3 g/day led to a reduction in the number of swollen joints, an improvement in the DAS28 score, and a decrease in CRP levels in patients with RA. In patients with AS and PsA, reduced pain and improved quality of life were observed [119,120,121,122,123].

Fiber, especially soluble fiber, present in vegetables, fruits, legumes, and whole grains, plays an important role in regulating the gut microbiota. The fermentation of fiber by probiotic bacteria leads to the production of SCFAs, such as butyrate, propionate, and acetate. SCFAs strengthen the intestinal barrier by reducing the permeability of the intestinal epithelium and limiting the translocation of lipopolysaccharides, which are potent stimulators of the inflammatory response [124].

Butyrate, as a key SCFA, inhibits the activation of the NF-κB pathway and enhances the function of regulatory T cells (Tregs), which reduce autoimmune reactions [125]. A diet rich in fiber may also support SCFA production in patients with PsA, which reduces both skin and joint lesions [63,126,127].

Emerging evidence indicates that high-fat diets (HFDs) can exacerbate the severity of rheumatic diseases by promoting gut dysbiosis and systemic inflammation. HFDs are associated with reduced microbial diversity and an overgrowth in proinflammatory bacterial species, such as *Proteobacteria* and *Firmicutes* subgroups, alongside a decline in beneficial commensals like *Faecalibacterium prausnitzii*. This dysbiotic shift compromises intestinal barrier integrity, leading to increased translocation of bacterial endotoxins (e.g., lipopolysaccharides) into the circulation, which activates Toll-like receptor signaling and amplifies inflammatory cytokine production. In animal models of rheumatoid arthritis and psoriatic arthritis, high-fat diets have been shown to worsen joint inflammation, cartilage destruction, and systemic markers of oxidative stress. Furthermore, the metabolic consequences of HFDs, including increased adiposity and insulin resistance, further fuel the inflammatory cascade by stimulating adipokine and cytokine secretion from adipose tissue. Thus, dietary patterns characterized by high fat intake may not only impair gut homeostasis but also potentiate immune dysregulation in rheumatic diseases [128].

**Figure 1:**
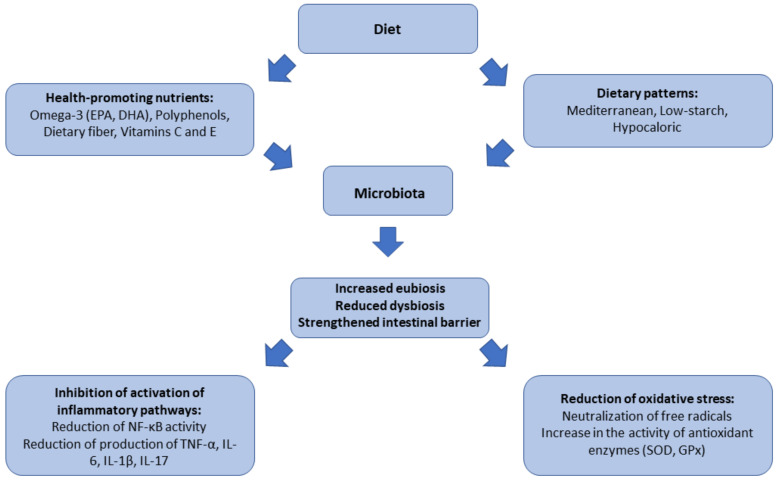
Interactions between diet, gut microbiota, and inflammation in autoimmune diseases.

Polyphenols are natural plant compounds with strong anti-inflammatory and antioxidant properties. They are found in large amounts in fruits, vegetables, tea, wine, and olive oil. The most active polyphenols, such as resveratrol, hydroxytyrosol, catechins, and quercetin, modulate signaling pathways related to inflammation, including NF-κB, AP-1, and Nrf2 [4,5]. Resveratrol, present in grapes and red wine, inhibits the activation of NF-κB and reduces the expression of genes encoding proinflammatory cytokines such as TNF-α, IL-1β, and IL-6. In animal models, resveratrol reduced joint damage in RA by inhibiting ROS production and reducing inflammation in the synovial membrane. Hydroxytyrosol, a key polyphenol of olive oil, has the ability to neutralize ROS and inhibit the activation of Th17 lymphocytes [129,130,131]. A comparison of Mediterranean and hypocaloric plant-based diet is presented in Table 2. Catechins present in green tea improve the function of endothelial cells, which is important in the context of reducing the risk of cardiovascular complications, which often occur in rheumatoid diseases [132,133]. Vitamins C and E, as key antioxidants, play an important role in neutralizing ROS and protecting cellular structures from oxidative damage [134]. Vitamin C, found in citrus fruits, berries, and vegetables, is a potent electron donor that reduces ROS, such as superoxide anions and hydrogen peroxide. It also increases the activity of antioxidant enzymes, such as superoxide dismutase (SOD) and glutathione peroxidase (GPx) [135,136]. Vitamin E, found in nuts, seeds, and vegetable oils, protects cell membranes from lipid peroxidation, which is an important protective mechanism in rheumatoid diseases. In clinical studies, vitamin E supplementation led to a reduction in disease activity in patients with RA [137]. The combination of vitamins C and E has been shown to synergize in protecting against oxidative stress, which may be particularly beneficial in AS and PsA, where ROS play a key role in pathogenesis. A summary of the influence of diet on microbiota and inflammation is shown in Figure 1.

Recent evidence suggests that dietary interventions, particularly anti-inflammatory and Mediterranean diets, may synergistically enhance the therapeutic effects of conventional pharmacological treatments such as NSAIDs and DMARDs in rheumatoid arthritis (RA) management. Anti-inflammatory diets, rich in omega-3 fatty acids, fiber, and probiotics, have demonstrated the potential to lower disease activity by reducing systemic inflammation, which could complement the immunosuppressive actions of DMARDs. Similarly, adherence to a Mediterranean diet may positively influence inflammatory markers and gut microbiota composition, potentially augmenting the efficacy of pharmacologic agents and improving overall patient outcomes. Therefore, integrating targeted nutritional strategies alongside standard drug therapy may offer a more comprehensive approach to controlling RA progression and symptoms [139,140].

## 11. Perspectives and Challenges in the Use of Diet Therapy in Rheumatoid Diseases

In recent years, diet therapy has gained importance as an adjunct to standard treatment methods for rheumatoid diseases such as RA, AS, and PsA. However, despite the growing evidence supporting the benefits of appropriate dietary patterns, there are still many challenges related to their implementation in clinical practice. One of the key challenges in diet therapy for rheumatoid diseases is the need to personalize dietary recommendations. Each patient is characterized by individual characteristics, such as the diversity of the gut microbiota, genetic polymorphisms related to nutrient metabolism and comorbidities that may affect the effectiveness of nutritional interventions. An example is the diverse response of patients with AS to a low-starch diet, which may be more effective in people with an excess of Klebsiella pneumoniae in the gut microbiota. In turn, in patients with PsA, a hypocaloric diet may bring the greatest benefits in the case of coexisting obesity or metabolic syndrome. Personalization requires the identification of biomarkers of response to dietary interventions. Examples of biomarkers include fecal SCFA levels, intestinal permeability indicators, and inflammatory markers such as CRP and IL-6. Modern methods such as microbiome analysis are also becoming increasingly important, allowing for the assessment of the composition of the gut microbiota and the identification of potential therapeutic targets. Another challenge is the lack of appropriate education on the role of diet in the treatment of rheumatoid diseases. Many patients are not aware of the potential benefits of changing their eating habits, and medical professionals often do not have sufficient knowledge about dietary therapy. The introduction of educational programs for patients could increase their involvement in the treatment process and improve the results of therapy. The education of specialists, including rheumatologists and dietitians, is equally important. Training on the mechanisms of action of dietary components such as omega-3, fiber, and polyphenols, and their impact on inflammatory and oxidative processes, could help to more effectively implement dietary recommendations in everyday clinical practice. Although there are many studies confirming the short-term benefits of dietary interventions, there is still a lack of long-term clinical trials assessing their durability and safety. Most of the available studies focus on specific dietary components or short-term interventions, which limits the generalizability of the results. For example, the effectiveness of the Mediterranean diet in reducing the long-term progression of joint damage in RA remains insufficiently documented. These studies should also consider the impact of dietary interventions on comorbidities such as cardiovascular disease, obesity, and diabetes, which are common complications of rheumatoid diseases. In addition, a better understanding of the mechanisms of action of dietary therapy is necessary, which may require the use of modern techniques such as metabolomics or microbiome analysis. Diet should be viewed as a complement to pharmacological therapy, not a substitute for it. Optimizing treatment requires cooperation between different specialists, including rheumatologists, dietitians, and psychologists. Dietary interventions can increase the effectiveness of disease-modifying antirheumatic drugs (DMARDs) and reduce the risk of adverse effects. For example, a diet rich in omega-3 may enhance the effects of biologics such as TNF-α inhibitors by additionally modulating inflammatory pathways.

Dietary changes can be difficult to implement due to cultural differences, dietary habits, and economic constraints. The Mediterranean diet, despite its proven benefits, can be difficult to implement in regions where access to fresh fruits, vegetables, and fish is limited. In such cases, it is necessary to adapt recommendations to local conditions and patient preferences, taking into account available resources.

## 12. Conclusions

Rheumatoid diseases, such as RA, AS, and PsA, are a serious health challenge in which inflammatory and oxidative processes play a key role in pathogenesis. Diet, as an environmental factor, plays an important role in the modulation of these processes, which makes it a promising tool supporting pharmacological therapy.

The importance of the Mediterranean diet in reducing inflammation and oxidative stress is well documented, as are the benefits of limiting starch in the diet of patients with AS or using a hypocaloric diet in PsA. Diet components such as omega-3 acids, fiber, polyphenols, and antioxidant vitamins have a beneficial effect on the gut microbiota, inflammatory pathways, and intestinal barrier function, which contributes to improving disease activity and quality of life of patients.

Despite the many benefits of diet therapy, there is a need for further research to optimize and personalize it. The education of patients and specialists, long-term clinical trials, and integration of diet therapy with pharmacological treatment are key to its effective implementation. In the future, diet may become an integral element of individualized care for patients with rheumatoid diseases.

## 13. Limitations

Although our review highlights important dietary and microbiota-related mechanisms involved in modulating inflammation in rheumatic diseases, some limitations must be acknowledged. Firstly, the complexity of immune responses in rheumatoid arthritis, ankylosing spondylitis, and psoriatic arthritis involves multiple cell types and signaling pathways beyond those predominantly discussed, such as γδ T cells contributing to IL-17 production. Secondly, while reactive oxygen species (ROS) are often associated with tissue damage and chronic inflammation, low levels of ROS can exert immunoregulatory functions by neutralizing reactive nitrogen species (RNS), a nuance that was initially underemphasized. Furthermore, most evidence stems from observational studies, animal models, and relatively short-term dietary interventions, limiting the generalizability to long-term human disease outcomes. Lastly, dietary patterns and gut microbiota composition are influenced by numerous environmental, genetic, and lifestyle factors that introduce variability in responses to nutritional interventions. Future research should aim to better delineate these complexities and integrate personalized approaches in the context of diet therapy for rheumatic diseases.

## Figures and Tables

**Table 1:** The influence of selected gut microbiota on RA, AS, and PsA.

Microbiota	Impact on Disease	References
*Prevotella copri*	RA—inflammatory induction through the activation of Th17 lymphocytes and IL-17 production; possible cross-reaction with autoantigens.	[53,59,74]
*Escherichia coli*	PsA—LPS translocation, IL-23/IL-17 pathway activation; increase in proinflammatory cytokines.	[37,73]
*Klebsiella pneumoniae*	AS—activation of IL-23/IL-17 pathway; stimulation of IL-17 production.	[65,66]
*Faecalibacterium prausnitzii*	Common for RA, AS, and PsA—SCFA production, anti-inflammatory activity, enhancement of intestinal barrier.	[53,59]
*Collinsella aerofaciens*	RA—increased intestinal permeability, induction of inflammation, activation of NF-κB.	[53,58]
*Lactobacillus salivarius*	RA—reduction in proinflammatory cytokines, increase in the number of Treg lymphocytes.	[36,69]
*Lactobacillus plantarum*	RA—reduction in disease activity, reduction in IL-17 and TNF-α.	[36,53]
*Roseburia faecis*	RA—anti-inflammatory effect, reduction in RF and ACPA, improvement of the intestinal barrier.	[75,76]
*Akkermansia muciniphila*	RA—improvement of the intestinal barrier, anti-inflammatory effect, reduction in intestinal permeability.	[69,77]
*Eggerthella lenta*	RA—induction of inflammation, increased activity in proinflammatory cytokines.	[58,78]

**Table 2:** Comparison of the Mediterranean diet and plant-based hypocaloric diet: nutritional profiles and anti-inflammatory potential [138].

Aspect	Mediterranean Diet	Plant-Based Hypocaloric Diet
Main Components	Vegetables, fruits, whole grains, olive oil, fish, nuts, and small amounts of dairy and red meat	Vegetables, fruits, legumes, whole grains, nuts, and seeds
Protein Sources	Fish, poultry, legumes, and limited dairy	Legumes, tofu, tempeh, nuts, and seeds
Fat Sources	Olive oil, nuts, and fish	Nuts, seeds, and plant oils (e.g., flaxseed oil, canola oil)
Fat Profile	High monounsaturated fats and moderate omega-3 intake	Very high in polyunsaturated fats (plant-based omega-3)
Fiber Content	Moderate to high	Very high
Polyphenol Content	High (from fruits, vegetables, olive oil, and wine)	Very high (from a wide variety of plant foods)
Key Anti-Inflammatory Components	Olive oil (oleocanthal), omega-3 fatty acids from fish, and polyphenols	Polyphenols, fiber, phytochemicals (e.g., isoflavones)
Anti-Inflammatory Potential	High	Very high
Typical Limitations	Limited intake of red meat, sugars, and saturated fats	Excludes animal products (in vegan diet), and potential risk of B12, iron, and calcium deficiencies
